# Neglected Tropical Diseases in Italy: introducing IN-NTD, the Italian network for NTDs

**DOI:** 10.1017/S0031182023000422

**Published:** 2023-10

**Authors:** Adriano Casulli, Spinello Antinori, Alessandro Bartoloni, Stefano D'Amelio, Albis Francesco Gabrielli, Giovanni Gazzoli, Laura Rinaldi, Fabrizio Bruschi, IN-NTD network

**Affiliations:** 1 Department of Infectious Diseases, Istituto Superiore di Sanità, Rome, Italy; 2 European Union Reference Laboratory for Parasites (EURLP), Istituto Superiore di Sanità, Rome, Italy; 3 Department of Biomedical and Clinical Sciences, Università degli Studi di Milano, Milan, Italy; 4 III Division of Infectious Diseases, ASST Fatebenefratelli Sacco, Milan, Italy; 5 Italian Society for Infectious and Tropical Diseases (SIMIT), Prato, Italy; 6 Department of Experimental and Clinical Medicine, University of Florence, Florence, Italy; 7 Infectious and Tropical Diseases Unit, Careggi University Hospital, Florence, Italy; 8 Department of Public Health and Infectious Diseases, Sapienza University of Rome, Rome, Italy; 9 Department of Control of Neglected Tropical Diseases, World Health Organization, Geneva, Switzerland; 10 Italian Association Amici di Raoul Follereau (AIFO), Bologna, Italy; 11 Department of Veterinary Medicine and Animal Production, University of Naples Federico II, Naples, Italy; 12 Department of Translational Research, N.T.M.S., School of Medicine, University of Pisa, Pisa, Italy; 13 Italian Society of Parasitology (SoIPa), c/o Department of Public Health, Sapienza Università di Roma, Rome, Italy

**Keywords:** Italian network, neglected tropical diseases, parasitic diseases, public health

## Abstract

The World Health Organization (WHO) defines neglected tropical diseases (NTDs) as a diverse group of primarily infectious diseases, which disproportionately affect poor and marginalized populations worldwide. In this context, NTDs are responsible for important morbidity and mortality and justify a global response. Moreover, NTDs are relatively neglected by research and development as well as by funding, if compared with the magnitude of the public health problem they represent. This happens even though, unlike other infectious diseases, they can be prevented, controlled and eliminated by targeted public health interventions. NTDs are mainly prevalent in communities from low-income countries in tropical and sub-tropical areas but are also present in upper–middle-income countries, including several in Europe. Here, we provide an update on the most relevant parasitic endemic or imported NTDs in Italy and illustrate the rationale for the establishment of the Italian network on NTDs, an alliance of scientific societies, institutes, foundations, universities and non-profit organizations united to fight NTDs.

## Graphical abstract

**Figure:**
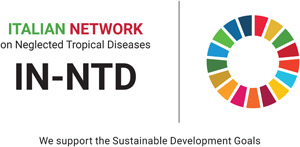

## Introduction

The use of the term ‘neglected tropical diseases’ (NTDs) represents a transition from the study of tropical diseases during the 19th century, particularly relevant in those countries with large colonial holdings, where tropical institutes were established, to one prioritizing the condition of neglected populations living in extreme poverty (Hotez, 2010). NTDs in fact are diseases that affect the poorest people (we should rather call them *diseases of neglected people*). Approximately 800 million (M) individuals live in extreme poverty (on <$1.90 day^−1^) globally, a large proportion of whom is infected with 1 or more NTDs (Molyneux *et al*., 2021). Many NTDs are vector-borne, food-borne or zoonotic. Despite being responsible for millions of cases a year globally, these diseases are often overlooked and forgotten, in terms of investments, research and political commitment. Although not widely known, they nevertheless cause the loss of 19 M disability-adjusted life years each year, an impact comparable to that of well-known diseases such as meningitis. Approximately, 1.7 billion (B) people need a treatment each year for 1 or more NTDs. Taken individually, many NTDs may not be perceived as a public health priority, but when considered together, their burden is not much lower than that of each of the 3 ‘big killers’ (tuberculosis, human immunodeficiency virus/acquired immunodeficiency syndrome and malaria) prioritized during the United Nations General Assembly Special Session in 2001 (Gruskin, 2002; Hotez *et al*., 2014). People affected by NTDs often live in rural areas and distant from health facilities. Although mainly endemic resource-poor countries in tropical and sub-tropical regions, NTDs are also found in industrialized countries located in temperate climatic zones (Hotez, 2018), including Italy (Piseddu *et al*., 2017; Gianchecchi and Montomoli, 2020).

The aim of this paper is to summarize presentations and discussions which took place during a symposium held at the XXXII National Congress of the ‘Italian Society of Parasitology’ (SoIPa; https://www.soipa.it/), in June 2022 (Naples). The symposium had the dual purpose of reviewing epidemiology of NTDs in Italy, with a focus on the parasitic infections currently or formerly endemic in Italy, and was also the occasion to present to a wider audience the ‘Italian network on NTDs’ (IN-NTD), a newly established coalition of institutions dedicated to fighting NTDs.

## Symposium on the Italian network on NTDs

### Global prospective on WHO and NTDs

The World Health Organization's (WHO) work on NTDs is coordinated by the department of the same name which was established in 2005 (https://www.who.int/teams/control-of-neglected-tropical-diseases/overview). Its main responsibilities are normative (developing guidelines, setting norms and standards), advocacy (raising NTDs' profile in global health agendas) and country support. Today, WHO focuses on 20 NTDs, caused by parasites (helminths, protozoa as well as ectoparasites), bacteria, viruses, fungi and toxins (https://www.who.int/teams/control-of-neglected-tropical-diseases/overview). NTDs are a very diverse group of conditions that primarily affect resource-poor communities in tropical and sub-tropical countries. In 2016, WHO's Strategic and Technical Advisory Group for NTDs defined processes and criteria to add new NTDs to the WHO list. Additions included mycetoma (2016), chromoblastomycosis and other deep mycoses (2017), scabies (2017) and snakebite envenoming (2017).

In January 2021, WHO launched its second NTD road map (WHO, 2020). The document is meant to strategically guide NTD activities during this decade but can also be used as an advocacy and policy development aid, and as a stakeholders coordination tool. The road map sets 2030 targets and intermediate milestones for many overarching, cross-cutting and disease-specific indicators. It also identifies programmatic action required to reach such targets, promotes cross-cutting and cross-sectoral approaches and facilitates country ownership of NTD programmes.

Much progress has been made in reducing the global burden of NTDs. Between 2010 and 2021, the number of people requiring NTD interventions was reduced by 25%, from 2.19 to 1.65 B, while 47 countries have eliminated at least 1 NTD as of March 2023, 8 of which were acknowledged in 2022 and 1 in 2023. Nevertheless, NTD programmes were among the most frequently and severely disrupted by the coronavirus disease 2019 (COVID-19) pandemic (WHO, 2022*a*). In 2020, only 798 M people were treated for NTDs, compared to 1.21 B in 2019. Although this number increased to 894 M in 2021, much needs to be done to regain pre-COVID-19 levels (WHO, 2022*c*). Disruptions affected both community-based interventions such as preventive chemotherapy and active case-finding, and health-facility-based interventions (diagnosis, treatment and care including surgery). Despite the COVID-19 pandemic, advocacy activities conducted by the global NTD community in 2021 and 2022 included the establishment of the Group of Friends on Defeating NTDs at the UN Secretariat in New York (30 November 2021), the celebration of 30 January as World NTD Day (one of the global health days), the adoption of the Abu Dhabi Declaration on Eradication of Guinea Worm Disease (23 March 2022) and the launch of the Kigali Declaration on NTDs (23 June 2022). Efforts were also made to improve the arsenal of NTD medicines and diagnostic tools: 18 target product profiles for diagnostics have been published by WHO so far, and in 2021–2022, 2 formulations of praziquantel, 1 of albendazole and 1 of ivermectin, were added to the WHO list of 10 prequalified NTD medicines (WHO, 2022*d*). Several publications were released in 2021–2022, including guidelines on schistosomiasis and visceral leishmaniasis and several road map companion documents dedicated to sustainability, monitoring and evaluation, One Health, skin NTDs, Water, Sanitation and Hygiene (WASH) and investing for NTDs. WHO has also increased its offer of virtual resources by launching apps (for the road map and on skin NTDs), and by making online courses available on the Open WHO platform: as of March 2023, 20 different topics are accessible, most of them in different languages, for a total of 42 courses (WHO, 2022*e*). In general, significant progress has been made to tackle the burden of NTDs over the past decade. However, service disruptions caused by COVID-19 pose a serious risk of set-back and losing the gains made so far. Accelerated efforts are needed to keep NTD programmes on track and achieve the 2030 targets. Continued support of partners, networks and all stakeholders is essential.

### WHO collaborating centres on NTDs in Italy

WHO collaborating centres (WHO CCs) are entities such as research departments/laboratories of universities/hospital/institutes designated by WHO to conduct activities in support of its programmes. They serve as sources of information, services and expertise for WHO and its Member States, as well as resources for training, research and collaboration. The designation of a WHO CC both recognizes past collaboration with WHO and provides a formal framework for future joint activities. It is a time-limited collaboration agreement between WHO and the designated institution, whereby the latter commits to carry out a set of concrete activities, referred to as Terms of Reference (ToR), which are jointly agreed with WHO CCs officers. Official data on WHO CCs worldwide are available on the WHO CCs Database (https://apps.who.int/whocc/). Currently, there are over 800 WHO CCs in over 80 WHO Member States (https://www.who.int/about/collaboration/collaborating-centres), covering various areas of global public health, including NTDs.

**Figure 1.**
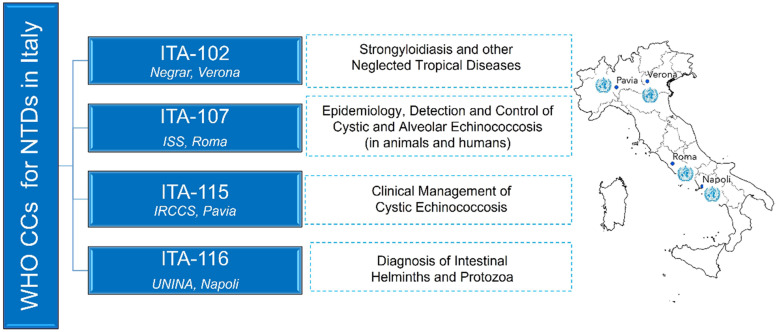
Four NTD collaborating centres in Italy designated by WHO.

Italy accounts for nearly 30 CCs, 4 of which are dedicated to NTDs (Fig. 1). Key activities of these 4 CCs include (a) improving the diagnosis, management and control of parasitic NTDs, such as soil-transmitted helminthiasis (STH), strongyloidiasis and cystic and alveolar echinococcosis; (b) monitoring the prevalence and burden of these diseases in endemic and non-endemic countries; (c) supporting endemic countries in research, education and services and (d) surveillance and health care for migrant populations. Existing partnerships among the Italian WHO CCs have proven successful in attracting funding, publishing joint reviews and opinion papers (e.g. Montresor *et al*., 2020; Rossi *et al*., 2020), and strengthening collaboration with WHO's NTD programmes. However, a further step is needed to reinforce existing synergies among the WHO CCs and explore new ones. WHO encourages its CCs to work more closely together to ensure the translation of research-based evidence into policy, practice and public health advancements. Based on these recommendations, 3 Italian CCs on NTDs have currently joined the IN-NTDs as well as the newly established Consortium of Experts in Neglected Tropical Diseases (CENTD; https://www.centd.org/en), an international thematic network coordinated by the WHO CC at Ghent University (Belgium).

### Focus on NTDs in Italy

While their impact is particularly heavy in Africa, Latin America and Asia, NTDs are nowadays seen with increasing frequency outside endemic regions, due to climate change, migrations and international travelling. Although a systematic surveillance for NTDs in Italy is not in place, and only few NTDs are notifiable by national health system, with an exception for imported arboviruses, rabies, leishmaniasis and scabies (which require compulsory notification), available data show that NTDs' burden in the country is far from being negligible.

According to a survey conducted in 9 Italian sentinel centres, schistosomiasis, strongyloidiasis and Chagas disease were the most common NTDs observed in a 7-year period (2011–2017) (Zammarchi *et al*., 2020*a*). Other documented NTDs from these selected centres were cystic echinococcosis (CE), leishmaniasis, cysticercosis, scabies and filariasis (Zammarchi *et al*., 2020*a*, 2020*b*). The number of cases showed an increasing trend, mainly driven by foreign-born subjects. A study aimed at providing an insight of the epidemiology of selected NTDs at national and regional level in Italy, based on hospital discharge records, retrieved 7195 hospitalizations during the period 2011–2016 involving individuals of Italian origin (60%) and foreign born (40%) (Tilli *et al*., 2020). In this study, the most common diagnoses were leishmaniasis (34% of selected NTDs; endemic and imported cases), schistosomiasis (29%; imported cases), strongyloidiasis (12%; imported cases), Chagas disease (8%; imported cases), dengue (8%; endemic and imported cases), taeniasis/cysticercosis (4.8%; imported cases) and filariasis (3%; imported cases) (Tilli *et al*., 2020). Hospitalization rates per 100 000 residents for all these selected NTDs were 2.05, 1.33 and 10.39 in the general population, Italians and foreign citizens, respectively. Hospitalization rates higher than 100 per 100 000 subjects were found in citizens from sub-Saharan Africa (SSA) and Bolivia (Tilli *et al*., 2020). It should be noted that from this study on selected NTDs, CE was excluded since it was elsewhere analysed with 12 619 patients (endemic but also imported cases) detected during the period 2001–2014 (Piseddu *et al*., 2017). All other NTDs prioritized by the WHO and not mentioned above are to be considered non-endemic in Italy, with the exception of several outbreaks of opisthorchiasis (foodborne trematode infections) and chikungunya (Pozio *et al*., 2013; Vairo *et al*., 2019).

All the data reported above are likely to underestimate the real prevalence in Italy, since most NTDs do not require hospitalization and several cases in illegal migrants might remain undiagnosed. These data suggest the need for education and training at university and post-graduate levels to increase the awareness of NTDs among healthcare professionals, as well as for targeted public health interventions (such as screening or presumptive treatment in high-risk groups) to improve clinical management and control of such diseases. For example, screening asymptomatic adult Latin American migrants for Chagas disease in Europe and treating *Trypanosoma cruzi*-seropositive individuals with antiparasitic therapy has been identified as a cost-effective strategy (Requena-Méndez *et al*., 2017), as is the case for serological screening of *Strongyloides stercoralis* infection in migrants from SSA arriving in Italy (Zammarchi *et al*., 2020*a*, 2020*b*). Recently, a study showed that presumptive treatment and screening strategies are more cost-effective than the current passive diagnosis for the public health management of schistosomiasis in SSA migrants to Italy, especially in the longer term (Zammarchi *et al*., 2023).

### Leishmaniasis in Italy

Italy is one of the countries in the Mediterranean basin traditionally considered endemic for leishmaniasis, a neglected vector-borne zoonotic disease caused, in this area, by *Leishmania infantum*, responsible for both cutaneous and visceral forms, with dogs recognized as the main reservoir. According to data reported to WHO, less than 100 cases of both human cutaneous (CL) and visceral leishmaniasis (VL) were reported in Italy in 2020 (Ruiz-Postigo *et al*., 2021), most of which were autochthonous. However, although case notification for human leishmaniasis is mandatory in Italy, the disease is generally underreported, especially CL. A recent study to identify the spatio-temporal and meteo-climatic patterns of VL among Italian citizens using the national Hospital Discharge Register (HDR) confirmed that the highest excess risk for the disease was observed in the major islands (i.e. Sicily and Sardinia) and in some area of southern Italy (Moirano *et al*., 2022). However, high standardized incidence ratios were also observed in some coastal and inland areas of northern Italy, confirming the northward expansion of vector populations due to climatic changes. This is consistent with the detection of new endemic foci of canine and human leishmaniasis in our country (Moirano *et al*., 2020; Gaspari *et al*., 2022; Gradoni *et al*., 2022). The study by Moirano *et al.* showed, during an 8-year period (2009–2016), 1126 cases of VL with a crude incidence rate (IR) of 2.43 cases per 10^6^ person-years with 2 age peaks: the first in the age groups of 0–4 years (IR 7.63 cases) and the second in the age group of 65–74 years (IR: 2.79 cases) (Moirano *et al*., 2022). The highest IR (16 cases per 10^6^ person-years) was reported in the Agrigento province of Sicily; these data agree with a retrospective study of leishmaniasis conducted in Sicily from 2013 to 2021 that showed that the largest number of cases of human and canine leishmaniasis were from Agrigento (45.4 and 41.5%, respectively) (Bruno *et al*., 2022; Moirano *et al*., 2022). The changing epidemiologic landscape for leishmaniasis in Italy with the recognition of new endemic foci is further confirmed by the upsurge (observed since 2012) in VL and CL cases diagnosed in the Emilia-Romagna region (Varani *et al*., 2013; Gaspari *et al*., 2022). However, in the same period, no rise in *L. infantum* infection among dogs was observed and molecular typing studies clearly showed that the protozoan strains circulating in dogs belonged to different population in comparison with the strains coming from human cases and sand flies (Rugna *et al*., 2017, 2018). Furthermore, the biting preference of *Phlebotomus* (Ph) *perfiliewi*, the suspected vector responsible for the upsurge of human cases in Emilia-Romagna, suggests the presence of a sylvatic reservoir other than dogs able to maintain and disseminate the infection in such areas (Calzolari *et al*., 2019). New epidemiologic studies conducted in Central Italy and the Pelagie Islands (between Sicily and Tunisia) show the detection of *L*eishmania *tarentolae*, historically considered a non-pathogenic protozoan of reptiles, in humans, healthy sheltered dogs and sand flies raising the question about its interaction with *L. infantum* (Iatta *et al*., 2021). Finally, from a clinical point of view, the increasing frequency of muco-cutaneous presentation of *L. infantum* among immunocompromised hosts (Simon *et al*., 2011) and the observation of post-kala-azar dermal leishmaniasis also in the Mediterranean basin in the same population (Antinori *et al*., 2007) should be mentioned.

### CE in Italy and Europe

Both cystic and alveolar echinococcosis are considered NTDs by WHO (Casulli, 2021; WHO, 2021). Within NTDs, CE caused by *Echinococcus granulosus sensu lato* (*s.l.*) complex and alveolar echinococcosis caused by *Echinococcus multilocularis* are considered the most relevant food-borne parasitic diseases both in Italy and in Europe (Bouwknegt *et al*., 2018). CE affects mainly pastoral and rural communities in both low-income and upper–middle-income countries; in Europe, it should be regarded and managed as a rare and orphan disease. Human CE is a notifiable parasitic infectious disease in most European countries and generally addressed by the European Union (EU) regulations. In practice, CE is largely under-reported by national health systems, which leads to a major uncertainty on the real burden of this zoonotic disease on European national health systems. Recently, a study conducted by means of a systematic review approach generated a quantitative model aimed at decreasing the limit of uncertainty on the public health burden of human CE in Europe (Casulli *et al*., 2022*a*). This study, using a multisource data approach at national level, identified around 65 000 human cases from 40 European countries during the period 1997–2021. It also identified a mean annual incidence of 0.64 cases per 100 000 people in Europe during the same period. According to this European study, 2 main public health hot-spot areas were recognized in Europe: the Mediterranean and the south-eastern European countries. While incidences and trends are decreasing in the Mediterranean area (e.g. in Italy and Spain), they remain stable or increasing in the southern Baltic area and in the Balkan Peninsula, in particular in Lithuania, the Republic of North Macedonia and Serbia. Based on incidence and trends from this study, the epicentre of CE in Europe is currently represented by the Balkan Peninsula. Even if Italy reported 0 cases of CE during the period 2012–2021 to The European Surveillance System (TESSy), this zoonotic disease is an unrecognized and neglected clinical condition in this country, since southern Italy and its major islands should be considered one of the most endemic area for CE in Europe (Piseddu *et al*., 2017). In fact, multisource data approach, mainly based on hospital discharge records, revealed more than 15 000 human CE cases and around 25 000 hospitalizations during the period 1997–2021 (Casulli *et al*., 2022*a*).

Another study conducted at European scale by means of a systematic review approach identified 597 human molecularly confirmed cysts caused by *E. granulosus s.l.* (Casulli *et al*., 2022*b*). According to this study, human infections in Europe are mainly caused by *E. granulosus sensu stricto* (*s.s.*) (G1 and G3 genotypes; 77.05%), secondarily by *Echinococcus canadensis* cluster (G6/7 and G10 genotypes; 21.77%), but also by *Echinococcus ortleppi* (G5 genotype; 1.18%). The approximate geographical distribution of these 3 species in Europe was identified as the Southern and South-eastern Europe for *E. granulosus s.s.*, the Central and Eastern Europe for *E. canadensis* (G6/7) and Central and Western Europe for *E. ortleppi*. Italy, representing 8.04% of this European sampling, recorded 100% of *E. granulosus s.s.* in humans.

### Geohelminthiases in Italy

Geohelminthiases or STH are the most widespread parasitic diseases in the world, with over 913 million children requiring preventive chemotherapy with benzimidazoles (WHO Global Health Observatory). The incidence of STH is particularly high in tropical and sub-tropical areas where poverty and poor hygiene conditions may favour their persistence (Bruschi, 2023). Many parasitic nematode species are co-endemic and mixed infections are frequently observed in a single individual, due to similarities in the transmission routes, environmental and socio-economic conditions and geographical distribution. Although Italy is no longer considered endemic for STH, that was not the case until a few decades ago, with instances of hyper-endemism and micro-epidemic events being reported as late as the 20th century.

It is well known that *Ancylostoma duodenale* was first described by Angelo Dubini in Milan in 1843. At the end of the 19th century (1880s), Italy faced one of the main epidemic *events* due to STH, namely the outbreak of ancylostomiasis affecting hundreds of miners during the construction of the San Gottardo tunnel. The detection of its aetiological agent by Grassi, Parona and Perroncito paved the way for the understanding of one of the first recognized occupational health threats in Europe (Parona, 1880; Perroncito, 1909). After the Second World War, poor hygienic conditions in rural areas of Southern Italy were identified as drivers of diffuse intestinal parasitism. In 1960, a congress focused on the epidemiological situation of the Sicilian village of Palma di Montechiaro, where the incidence of intestinal nematodiasis was astonishingly high, was chaired by Carlo Levi (a well-known writer and artist who was also a physician) and organized by Danilo Dolci (a sociologist deeply involved in the fight against mafia) and Silvio Pampiglione (a parasitologist and tropicalist from the University of Bologna). Following the congress, a multidisciplinary plan of development was established, leading to a substantial decrease of the public health impact of parasitic diseases in the area. Few years later, Giovanni Berlinguer, Lia Paggi and Paola Orecchia (from the Sapienza University of Rome) reported the occurrence of nematode infections in school-age children from the surroundings of Rome and the impact of parasitism on learning performances and school grades (Berlinguer *et al*., 1964). In the following years, the generally improved hygienic and social conditions in Italy led to a substantial decrease of the incidence of nematodiases, which are now mostly diagnosed in people originating from tropical countries. However, sporadic events are recorded, mostly due to the zoonotic transmission of members of the *Ascaris* and *Strongyloides* genera. The genus *Ascaris* contains 2 species, namely *Ascaris lumbricoides*, a parasite of humans and *Ascaris suum*, which infects pigs. Although mainly found in different hosts, the 2 species are morphologically very conservative, with little or no variation in morphological traits. Evidence from molecular studies suggests the presence of gene flow between the 2 species and their different occurrence seems to be based on the endemicity of the human species. In Italy, where *A. lumbricoides* is not endemic, most of the human infections are nowadays zoonotic and *A. suum* is the main causative agent of ascariasis (Cavallero *et al*., 2021).

As for *S. stercoralis*, human infections tend to be acute, sometimes evolving to chronicity while in immunocompromised patients they may become widely disseminated up to be fatal. Having *Strongyloides procyonis* as a closest taxon to *S. stercoralis* may indicate that the 2 species share a common ancestor, and it might be assumed that *S. stercoralis* originally evolved as a parasite of canids, and later spread into humans as a result of dog domestication. Epidemiological data suggest that *S. stercoralis* type A can infect both humans and dogs while type B has not adapted to infect humans; this might be the ancestral phenotype of this species. Therefore, the zoonotic potential of this species and its occurrence in Italian dogs is worth being investigated and monitored (Tamponi *et al*., 2020).

### Non-parasitic NTDs in Italy: leprosy

Leprosy is an NTD caused by *Mycobacterium leprae*, which develops in susceptible individuals and predominantly affects the skin and peripheral nerves. The disease is curable and treatment in the early stages can prevent disability. In Italy, leprosy is included among the rare diseases with exemption code RA0010 (‘Hansen's disease’). It can occur anywhere as an imported disease, i.e. in Italians who have stayed in endemic countries and in migrants from such countries: between 6 and 9 cases are diagnosed each year. The most frequent clinical forms are borderline tuberculoid, borderline lepromatous and lepromatous leprosy (Nunzi and Massone, 2009). The autochthonous outbreaks that occurred in Calabria, Liguria, Apulia, Sardinia and Sicily during the last century are practically extinct, with an average of 1 autochthonous case every 10 years. From a regulatory point of view, the control of leprosy is based on the Presidential Decree of 21 September 1994, known as the ‘Act of Guidance and Coordination to the Regions and Autonomous Provinces on Hansen's disease’, which established 4 National Reference Centres in Italy for leprosy treatment located in Cagliari (Sardinia), Genova (Liguria region), Gioia del Colle (Apulia) and Messina (Sicily). The reference centres are responsible for confirming the diagnosis and treatment, as set out in the Decree of the President of the Council of Ministers of the Italian Republic (DPCM) of 31/05/2001 (G.U. no. 182 of 7/08/2001).

When in a health unit of the National Health System a case of leprosy is suspected, the person must be referred to one of the 4 National Reference Centres for diagnostic confirmation and treatment. Worldwide data on leprosy are published annually in the Weekly Epidemiological Record (WER) of the WHO (WHO/WER – WHO, 2017*a*). The annual total number of new cases, after a significant decrease in the first 5 years of this century, remains almost stable (about 200 000 new cases each year), but over the past 3 years there has been some major variability, due to the impact of COVID-19 on data collection and implementation of control programmes. In 2021, there were 140 594 cases (of which 14 in Europe), higher than in 2020 (128 405 and 27 in Europe), but lower than in 2019 (202 185 people and 42 in Europe). The 3 countries with the largest annual number of new cases are India, followed by Brazil and Indonesia, which accounted in 2021 for 74.5% of the caseload globally. Other countries with a high number of new cases (>1000) are Bangladesh, R. D. of Congo, Ethiopia, Madagascar, Myanmar, Nepal, Nigeria, Philippines, Sri Lanka and Tanzania (WHO, 2017*b*). In the world, a high number of people, despite being treated clinically, live with permanent disabilities caused by the disease, and need physical rehabilitation. At the same time, the stigma still associated with the disease remains a barrier to interrupting transmission and has an important impact on peoples' lives, long after they have been cured. Considering all these factors, one can conclude that leprosy is still a public health problem in various countries of the tropical and sub-tropical belt.

### The rationale for establishing IN-NTD

Driven by the new roadmap 2021–2030 launched by WHO on 28 January 2021, it was decided to build up in our country a coalition of scientific societies, institutes, foundations, universities and non-profit organizations (ONLUS), to support the pathway towards the last mile towards the control, elimination and eradication of these devastating, neglected, poverty-related and poverty-causing diseases. IN-NTD is a network aimed at pursuing interactions among various institutions in Italy concerned with NTDs at national and international level. The network is not meant to be a new scientific society to which individual scholars/researchers can belong. Rather, the aim of the network is to coordinate the work of many to achieve the synergy and authority necessary to propose and organize interventions useful for combating NTDs in Italy, as well as in those countries where NTDs are endemic.

IN-NTD has several objectives: (1) advocacy: to raise awareness among wide audiences, such as decision makers, politicians, entrepreneurs, volunteers, students and advocate for effective interventions that can reduce the impact of NTDs on populations; (2) training: to promote a greater and targeted educational and didactic offer in Italian universities, and opportunities for capacity building, training and education in endemic countries in collaboration with local institutions; (3) research: to stimulate basic, applied research, in particular for the development of new diagnostic tools and treatments as well as operational research that translates evidence into guidance, policy and practice; (4) international public health cooperation: to foster cooperation with endemic countries to improve prevention, diagnosis and treatment and make existing guidelines and policies more effective; (5) sustainable development goals (SDGs): to contribute to SDG 3.3 that aims to ‘end NTDs’ and associated conditions, such as social exclusion, stigma, mental illness, socio-economic inequality and to help achieve the fundamental human rights of health for all.

The network will work on various fronts: (1) mapping: conducting a thorough assessment of Italian institutions and individuals working on NTDs, inviting them to join and cooperate with IN-NTD; (2) inform and raise awareness among policy makers, and mobilize resources to be used in Italy and in the context of international cooperation; (3) stimulate the establishment of sound coalitions between universities and research institutes to increase knowledge and pursue innovative interventions; (4) foster dialogue with the pharmaceutical and biotechnological industries promoting research, development, production and access to ready-to-use tools for countries that need them; (5) promote close collaboration with WHO and other international and multilateral agencies thus mainstreaming IN-NTD's work within global activities. In doing so, IN-NTD wants to draw attention to these 20 priority diseases and facilitate the implementation of interventions that reduce their impact on global public health. Much has been done, but much remains to be done in terms of access to medicines, diagnostics, vaccines and safe drinking water, as well as in terms of implementation of vector control, veterinary public health and training of health personnel.

**Figure 2.**
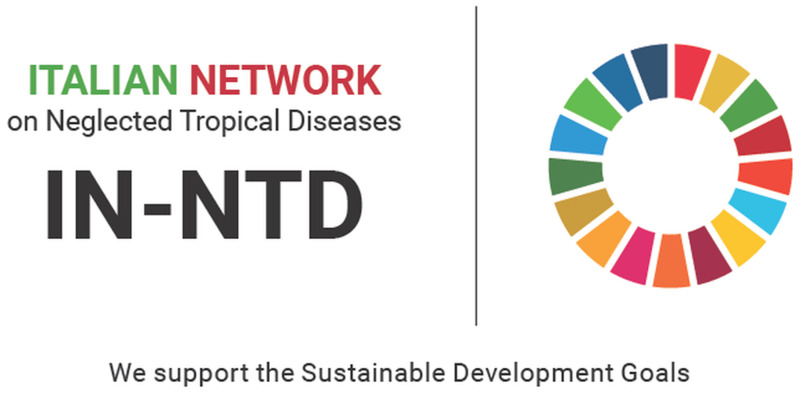
Logo of the ‘Italian network on neglected tropical diseases’ (IN-NTD).

Current participants on voluntary basis to IN-NTD are the Italian Society of Parasitology (SoIPA), Italian Society of Tropical Medicine and Global Health (SIMET), Italian Society of Infectious and Tropical Diseases (SIMIT), Istituto Superiore di Sanità (ISS), MACH Centre, University of Milan, IRCCS Sacro Cuore Don Calabria hospital, Ivo de Carneri Foundation (FIdC), Public Health Laboratory Ivo de Carneri (PHL-IdC), Sightsavers Italia Onlus, Italian Association Amici di Raoul Follereau (AIFO), Interuniversity Research Centre on Malaria – Italian Malaria Network (CIRM.IMN), IRCCS National Institute of Infectious Diseases L. Spallanzani (INMI) and UNESCO Chair in ‘Training and empowering human resources for health development in resource-limited countries’, – University of Brescia (Fig. 2).

IN-NTD is also aligned to the global response against these infectious diseases represented by national and international networks on NTD partners and platforms for advocacy and information-sharing, such as in Africa (African Research Network for NTDs, https://arntd.org/), Australia (The Leprosy Mission, https://www.leprosymission.org.au/leprosy/neglected-tropical-diseases/), Canada (Canadian Network for NTDs, https://cnntd.org/), France (Réseau Francophone sur les Maladies Tropicales Négligées, https://www.rfmtn.fr/), Germany (German Network against NTDs, https://dntds.de/the-network.html), Japan (Japan Alliance on Global NTDs, https://jagntd.org/), UK (Uniting to Combat NTDs, https://unitingtocombatntds.org/), USA (NTDS NGO Network, https://www.ntd-ngonetwork.org/ and Coalition for Operational Research on NTDs, https://www.cor-ntd.org/) and Switzerland (Swiss Alliance Against NTDs, https://santd.ch/home/about-us/?lang=en).

## Author's contribution

A. C. and F. B. conceived and designed the study. S. A., A. F. G., L. R., G. G., S. D'A. and A. B. conducted data gathering and wrote sections of the article. All the authors, including all the participants of the IN-NTD network, approved the last version of the manuscript.
